# Millisecond magnetic particle imaging of a cerebral aneurysm phantom with and without flow diverter stent

**DOI:** 10.1038/s44172-026-00726-0

**Published:** 2026-07-29

**Authors:** Olaf Kosch, Augusto Fava Sanches, Yigit Özpeynirci, Thomas Liebig, Frank Wiekhorst

**Affiliations:** 1 Department of Metrology for Magnetic Nanoparticles, Physikalisch-Technische Bundesanstalt, Berlin, Germany; 2 Department of Vascular Surgery, University Hospital LMU Munich, Munich, Germany; 3 Institute for Diagnostic and Interventional Neuroradiology, University Hospital LMU Munich, Munich, Germany

**Keywords:** Imaging techniques and agents, Imaging techniques, Biomedical engineering

## Abstract

Undetected, asymptomatic brain aneurysms are associated with risk of progressive expansion, followed by rupture and hemorrhage, increasing the risk of mortality and morbidity. Imaging techniques to assess the state and stent treatment of aneurysms are beneficial. By Magnetic Particle Imaging (MPI), we were able to resolve with millisecond temporal resolution the location and movement of a tracer bolus in different parts of an artery phantom derived from a patient specific CT data set including a cerebral aneurysm. To assess the success of a flow diverter stent treatment, we compared flow MPI measurements in the artery phantom without a flow diverter stent with images where a stent was inserted. Our results provide valuable information on the capabilities and prospects of MPI of vascular structures and the detection and quantification of flow changes in untreated and treated aneurysms for future in vivo applications.

## Abstract (web summary)

Olaf Kosch and colleagues show flow and flow changes caused by an aneurysm and by implanting a stent to direct the flow away from the aneurysm in a patient-specific cerebral artery phantom by Magnetic Particle Imaging and propose application scenarios.

## Introduction

Cerebral aneurysm is a disease characterized by local dilatation of the arterial walls in the intracranial vasculature, usually at arterial curves and bifurcations in the circle of Willis^1^. Such pathological weakness of an artery carries a high risk of progressive dilation with subsequent bursting or rupture of the artery, leading to a subarachnoid hemorrhage, which is associated with high mortality and morbidity^2^. Understanding the hemodynamic mechanisms involved is critical to identifying effective treatment options to reduce the risk of rupture and bleeding in cerebral aneurysms. An effective endovascular treatment is the use of a flow diverter stent^3,4^ to divert blood flow away from the aneurysm. Ideally, the blood flow in the aneurysm is stopped. The stagnation of blood flow leads to the formation of a thrombus within the aneurysm, resulting in its occlusion. In this paper, we show the capability of magnetic particle imaging (MPI) to detail the blood flow in an aneurysm and its changes through the presence of a flow diverter stent. With its high temporal and spatial resolution in detecting magnetic nanoparticles (MNP) employed as a tracer in a vascular system, MPI offers great potential for the analysis of hemodynamic situation of aneurysms. The capability of MPI for flow imaging was demonstrated recently in an internal carotid artery phantom with a realistic 3D aneurysm^5^ and compared with magnetic resonance imaging and dynamic digital subtraction angiography, where it was possible to track a MNP bolus through the model and to estimate the average flow velocity by MPI. However, it is difficult to discern further details of the hemodynamic flow due to the noise present in these MPI results. Though already a human brain MPI scanner^6^ exists for stroke studies, it only has a reduced suitability for hemodynamic imaging due to its low spatial and, especially, temporal resolution. The sensitivity and signal-to-noise ratio of our scanner have been enhanced by utilizing separate receiving coils and additional filters. The primary objective of this enhancement is to enable quantitative cell detection^7^. However, we also utilize this increased sensitivity for our hemodynamic imaging. The artifact-free MPI imaging of a straight vessel phantom to visualize the stent lumen in intracranial flow diverters was reported in ref. ^8^.

Flow diverter stents (FDS) are often used to treat large or giant aneurysms and wide-neck and fusiform aneurysms, where stent adaptation is a complex task with an overall complication rate of 17%^9^. In ruptured aneurysms, the complication rate rises to 30.6%. One of the main reasons for this high complexity is the lack of comprehensive hemodynamic data on the flow inside the aneurysm and the artery when fitting the FDS. In this study, we want to clarify whether MPI is sensitive enough to provide such detailed hemodynamic data and can help reducing the complexity of FDS adaptation. With this in mind, we analyzed the effect of an FDS placed in an aneurysm phantom by preclinical MPI on the hemodynamic flow conditions compared to the untreated aneurysm situation. By this, we want to assess the capability of MPI for imaging hemodynamic flow and flow changes caused by endovascular intervention. To this end, we imaged the dynamics of untreated and FDS-treated patient-specific cerebral aneurysms to demonstrate the benefit of MPI for endovascular treatment without accurately modeling the physiology of intracranial blood flow. To link high-resolution temporal and spatial MPI with patient data, we used 3D printing as a bridging technology.

## Methods

### Artery aneurysm phantom manufacturing

Two identical aneurysm phantoms were fabricated by additive manufacturing at the University Hospital of the LMU in Munich, Germany, based on CT data of a patient-specific internal carotid artery (ICA) aneurysm in the ophthalmic portion. The patient-specific aneurysm lumen geometry was reconstructed from contrast-enhanced CT imaging datasets acquired using an Integris Allura FD20 system (Philips Healthcare, Best, The Netherlands). The image volumes were reconstructed on an Xtravision Philips workstation within a 256 × 256 × 256 matrix and exported in DICOM format for further processing. The DICOM datasets were imported into OsiriX v5.6 (Pixmeo SARL, Bernex, Switzerland), where the region of interest (ROI) containing the aneurysm and adjacent parent vessels was selected. A preprocessing (“reparation”) step was performed to improve segmentation fidelity, including removal of acquisition-related artifacts, correction of discontinuities or spurious structures, and smoothing of the ROI to ensure a consistent representation of the inner lumen surface.

**Fig. 1:**
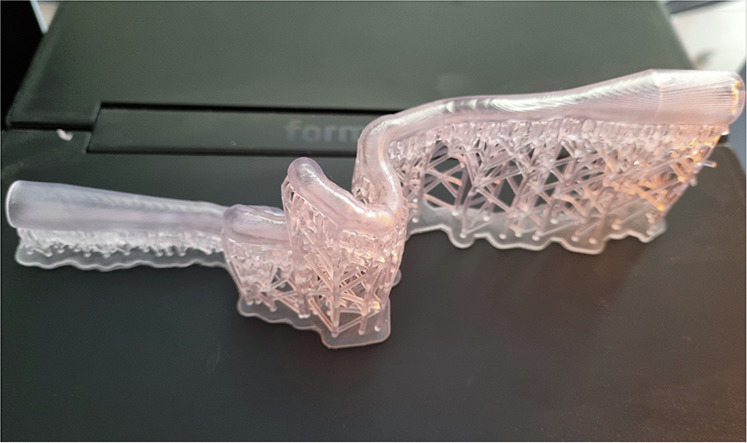
Cerebral artery phantom directly after printing by stereolithography with silicon-like resin. The printing support structure is still visible; the aneurysm itself is hidden by the support structure.

Following this preparation stage, the aneurysm lumen was segmented in OsiriX using intensity-based and semi-automatic tools, allowing separation of the contrast-enhanced blood pool from surrounding tissue. The segmentation results were iteratively refined to obtain a clean and continuous lumen volume. The final lumen segmentation was then converted into a triangulated 3D surface model, and the reconstructed geometry was exported in STL format. This STL model served as input for subsequent processing steps, including volume grid generation, phantom manufacturing, and computational analyses. The aneurysm was of saccular morphology (4.5 mm dome height, 6 mm cross-section, 4 mm neck, 3 mm parent artery diameter). The volume data of the phantom were printed on a stereolithography printer (Form 2, Formlabs GmbH, Germany) with a minimal layer height of 25 µm by use of silicon material (elastic 50 A resin, FormLabs GmbH, Germany). Fig. 1 shows one phantom just after printing so that the support structure still is visible. After that, the phantoms were first washed with isopropanol (Merck KGaA, Germany) using an ultrasonic bath and tempered at 50 °C, a cleaning step using a compressed-air gun followed. Finally, a post-cure procedure was carried out at 405 nm light wavelength and heating the phantoms at about 80 °C for 60 min. Then, the 3D printing supports were removed for better positioning during MPI measurements.

Into one of the two phantoms a flow divertering stent Pipeline Embolization Device (PED; Medtronic Neurovascular, Irvine, California, USA was catheterized to the aneurysm location and fixated by dilation.

### Measurement setup

**Fig. 2:**
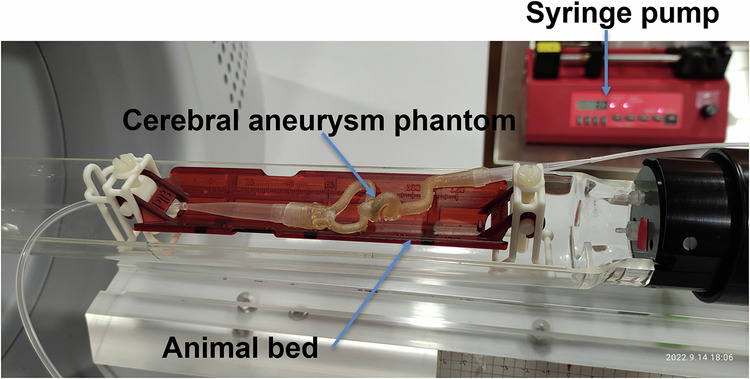
Vascular aneurysm phantom ready for MPI scanning. Before being inserted into the scanner for MPI measurements, the sample vascular phantom is connected to a syringe pump and filled with a mixture of glycerin and water.

The arterial phantom was placed on the animal bed and positioned inside the detection coils of the MPI scanner see Fig. 2. The vascular system of the phantom was connected to a syringe pump (LA-800, Landgraf Laborsysteme HLL, GER) to provide different, defined flow rates. In preparation of the experiments, the syringe, tubing, and phantom were filled with a glycerin-water mixture (59.1% glycerin, 40.9% distilled deionised water (ddH_2_O)) to mimic the viscosity of blood. The removal of air bubbles was accomplished by pumping the mixture through the tubes and the phantom. During the MPI data acquisition an MNP bolus of 100 µL Perimag (Micromod Partikeltechnologie, GER) at an iron concentration *c*(Fe) = 50 mmol/L was administered to the vascular phantom and pumped through it at a continuous flow rate of *Q* = 3 mL/min. The selection of this flowrate was made to illustrate the capacity of MPI to discern even minor turbulences. The dependence of the intensity of turbulences and vortices on the arterial flow rate is shown for untreated and treated aneurysms in ref. ^10^. The MNP bolus was prepared by placing it in a tube section outside the scanner. Prior to the initiation of the experiment, it was imperative to ensure that the bolus remained intact. Then afterwards, as the bolus passed through the cerebral artery and the aneurysm, the bolus was swirled and diluted in the medium by the passage.

### MPI parameter

All MPI measurements were performed using a preclinical MPI system (Bruker MPI 25/20 FF) installed at Charité University Hospital Berlin, equipped with an additional separate receiving coil to increase the sensitivity^11^ and additional receive filters (Bruker). This system is based on the movement of a field-free-point (FFP) through the whole imaging volume and employs the system function (SF) approach for image reconstruction^12^. A selection gradient field of Δ*B* = 1.2 T/m in the *z*-direction and Δ*B* = 0.6 T/m in *x*- and *y*-directions was used to generate the FFP and was implemented for all measurements presented in this work. By moving the FFP through the field of view (FOV), a spatially encoded signal of the whole imaging volume can be detected. The movement of the FFP on a closed 3D Lissajous-trajectory is accomplished by applying three orthogonal drive-fields oscillating at slightly different excitation frequencies at about 25 kHz (2.5 MHz divided by 102/96/99 in *x*-/*y*-/*z*-direction) with amplitudes of 12 mT. We chose these gradient and drive fields to obtain a sufficiently large physical FOV with a size of 40 × 40 × 20 mm^3^ to cover the arterial phantom without the inlet- and the outlet- sections. Thus, in case of a 3D acquisition scheme one total Lissajous-trajectory is completed after a period of 21.5 ms, allowing the reconstruction of a full 3D dataset of the FOV with millisecond resolution. The SF required for image reconstruction was recorded with a point-like MNP reference tracer with a volume of *V*_ref_ = 3 × 3 × 1.5 mm^3^ in a grid-like manner at various selected positions in the FOV. The SF was recorded using 100 averages and background measurement subtraction at a resolution of 45 × 27 × 15 voxels and 1.5 × 1.5 × 1.5 mm^3^ voxel size. The recording time of the SF was 18.5 h. The SF covered a volume of 67.5 × 40.5 × 22.5 mm^3^ with a comfortable overscan (see ref. ^13^) in the *x*- direction related to the size of the physical FOV, as the input and the output tubes of the arterial phantom were placed in this direction. For the MNP and the chosen voxel size, an image resolution of 3.7 mm was determined for *x*- and *y*- direction using the two-voxel analysis in ref. ^14^. In *z*-direction the resolution is better due to the higher gradient. The dynamic flow measurements of the arterial phantoms were performed using the same field parameter as the SF measurement, however without averaging to maintain 21.5 ms temporal resolution. At the beginning of each measurement, the background signal is recorded and removed from the recorded data in a processing step during the reconstruction. The reconstructions were performed using the Kaczmarz-algorithm with Tikhonov regularization in the ParaVision 6.0 MPI software (Bruker). The applied relative regularization factor was *λ*= 1·10^−5^. The frequency components used in the reconstruction process were selected by a minimum signal-to-noise-ratio (SNR) threshold based on the SNR calculated for all frequency components in the SF. An SNR threshold of 6 was applied and components below 40 kHz were discarded. All reconstructions were calculated with 20 Kaczmarz iterations to enforce high resolution imaging by a high number of iterations. To reduce the number of frames in the reconstruction from 4000 to 400, an averaging of 10 was applied for the phantom without the FDS because a longer observation time was needed to detect the late vortex flow in the aneurysm. We used an average factor of 2 in the experiment with FDS to reduce the number of time frames from 620 to 310. The reconstructed 3D time frames were exported to MIPAV, the Medical Image Processing, Analysis, and Visualization application (https://mipav.cit.nih.gov/). The utilization of MIPAV entails the rendering of the data set as a 4D sequence, thereby facilitating the visualization of the entire dataset, as opposed to merely individual cross-sections, with the objective of identifying all pertinent details. Therefore, a single image illustrates the rendered 3D data set of one reconstructed time frame of the MNP distribution. Please note that the rendered image does not permit quantitative analysis of the distribution because the 3D data has been reduced to a 2D image. For quantitative analysis, the data were read into MATLAB (MathWorks, Inc.), where a region of interest (ROI) close to the aneurysm was defined with a size of 7.5 × 7.5 × 4.5 mm^3^ for both cases (without and with FDS). The content of MNP within the ROI was plotted over time to visualize the amount of flow entering the aneurysm and the retention of MNP within the aneurysm.

### Reporting summary

Further information on research design is available in the Nature Portfolio Reporting Summary linked to this article.

## Results and discussion

With the knowledge from simulation studies, like in ref. ^10^ we know already that in a bulge like an aneurysm driven by the arterial main flow a vortex flow can exist. Together with MNP used as probes were capable to resolve the flow properties in the region of the aneurysm and the action of diverter stent in the aneurysm. Videos with millisecond resolution could be reconstructed from the MPI data. The subsequent images depict several of the key scenes from the videos; nevertheless, the videos offer a more exhaustive illustration of the hemodynamic flow. Video sequences are accessible under the following link (press download and open it in MS PowerPoint or OpenOffice): https://box.ptb.de/getlink/fiHDZn8LwhBTwejPCwZE86e4/aneurysm_videos.ppsx.

### Phantom without flow diverter stent

**Fig. 3:**
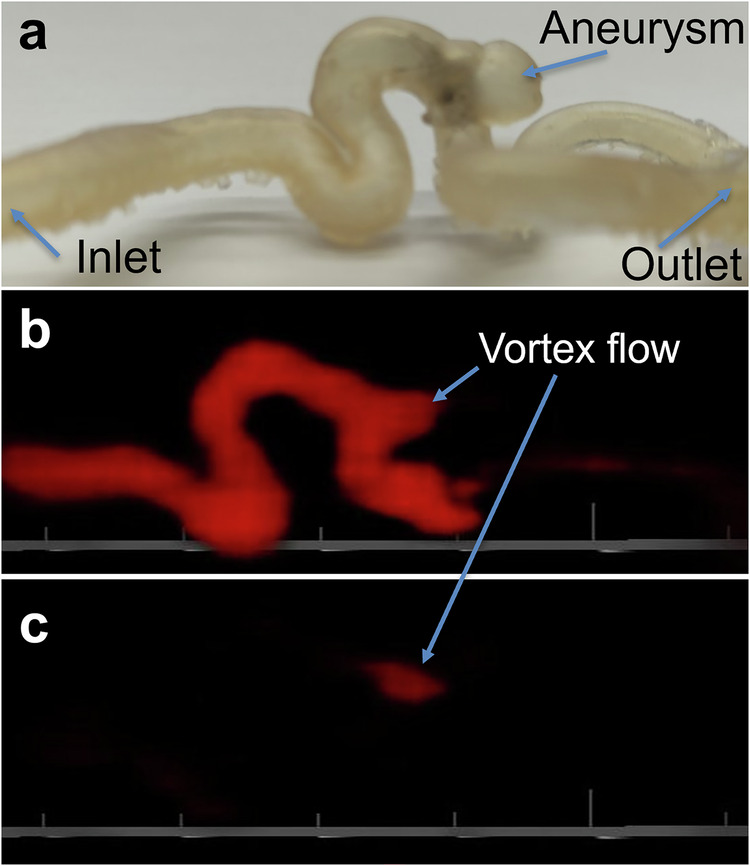
MPI of MNP bolus passage and vortex flow in the vascular phantom. In (**a**), the selected section of the phantom including aneurysm is pictured, in (**b**) the imaged section during passage of magnetic nanoparticles bolus, and (**c**) depicts the “late potential” of the vortex flow in the aneurysm.

Two typical reconstructed time frames of the arterial phantom without treatment by FDS are depicted in Fig. 3. For better comparison a photograph of the corresponding phantom section is shown in panel **a** of the figure. In panel **b** the passage of the MNP is shown at *t* = 9.6 s through the phantom while in panel **c** the late vortex flow at *t* = 63 s (an effect similar to the “late potentials” in MRI).

The aneurysm represents a bulge in the vessel wall in which the trapped hemodynamic volume is set in rotation by the tangential main flow in the artery, creating the vortex flow. As the MNP bolus passes through the artery, it is also flushed into the aneurysm. While the MNP bolus in the artery is then quickly transported away, the portion in the vortex flow of the aneurysm remains much longer.

**Fig. 4:**
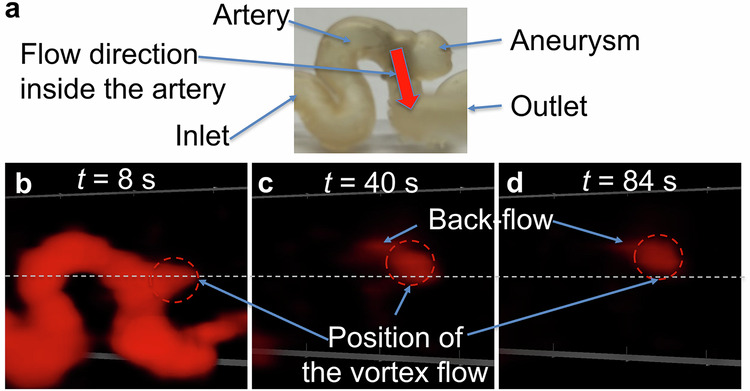
MPI resolves the MNP outflow from different regions of the art. In (**a**), the selected phantom section includes an aneurysm and **b**–**d** depicts the magnetic nanoparticles in the vortex flow inside the aneurysm at different time steps, in **b** at 8 s, in **c** at 40 s and in **d** at 84 s.

Figure 4 gives an enlarged view of the aneurysm area showing the flow dynamics recovered by MPI. Due to the direction of flow within the artery (see Fig. 4a), the MNP reached the lower part of the aneurysm first.

**Fig. 5:**
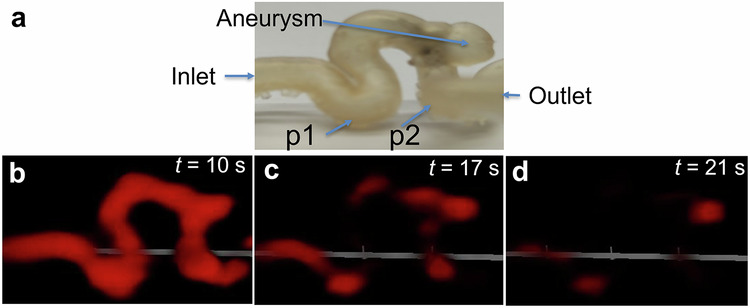
MPI resolves the MNP outflow from different regions of the artery. In (**a**), selected section of the phantom including the curvature regions p1 and p2, imaging of the outflow including weak vortex formations inside the curvature regions p1 and p2 in **b** at *t* = 10 s, in **c** at 17 s and **d** at 21 s.

Driven by the flow in the artery the vortex flow inside the aneurysm is changed from the bottom part (closer to the artery connection) to the upper part of the aneurysm. A dashed line has been added to the center of the panel **b**–**d** of Fig. 4 to clearly mark this relative change. In addition, the images at *t* = 40 s and *t* = 84 s (outside the red dashed circle) show a small backflow from the aneurysm back into the artery. This position of the backflow in the upper part of the aneurysm into the artery can then be explained by the vortex flow in the aneurysm. To facilitate a comprehensive understanding of these dynamic changes, it is recommended to refer to the video sequences that have been provided. In addition, we also can observe some differences in bolus outflow from different parts of the artery, where positions are marked in Fig. 5, and they are a function of the curvature and dilatation of the artery. Due to the limitations of the static image, we suggest viewing the first video sequence in the MS PowerPoint file. In areas of strong curvature and widening of the artery, such as p1 and p2 (see Fig. 5a), we also assume a weak vortex formation, but much more transient than in the aneurysm. The fact that we can image this with MPI at a relatively low flow rate of 3 mL/min demonstrates the high sensitivity of this imaging modality for mapping hemodynamic flows. The simulations of the arterial flow of untreated and treated aneurysms in ref. ^10^, impressively show the changes in hemodynamic flow due to treatment with an FDS, but also that the vortices and turbulence increase with increasing arterial flow rate, and this amplification should also make them easier to detect.

### Phantom with flow diverter stent

**Fig. 6:**
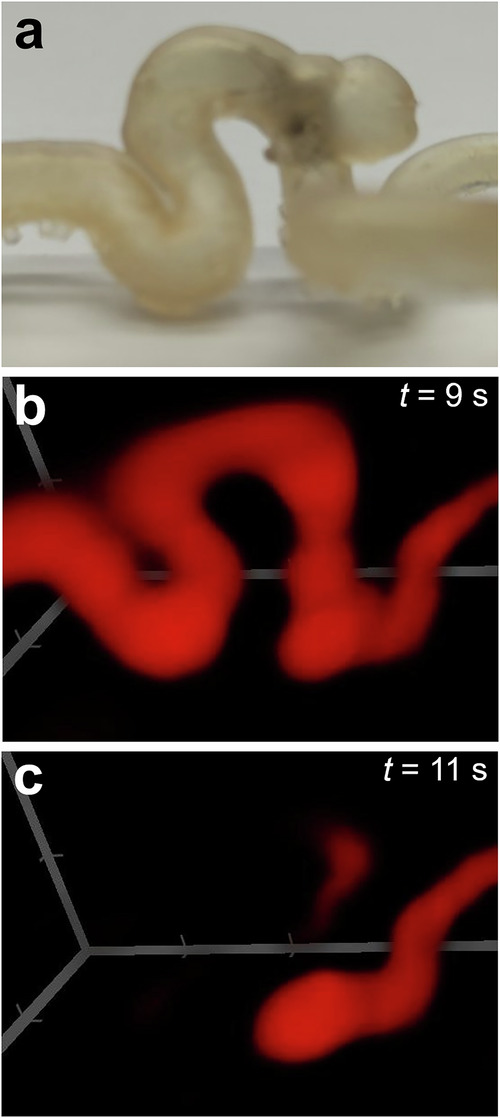
Strongly reduced vortex of the MNP in the arterial phantom with flow diverter stent. In (**a**), the phantom section is shown and the transport of the magnetic nanoparticles in **b** at 9 s and **c** at 11 s after the start.

In the aneurysm phantom with FDS, the MPI reconstructions reveal that the MNP much faster pass the aneurysm region, as if there was no vortex flow to observe in the aneurysm. Fig. 6 shows the MNP passage at two early time points, *t* = 9 s and *t* = 11 s, with the same selected section as in Fig. 4, but with the FDS. In contrast to the results for the aneurysm without stenting shown in Figs. 3 and 4, we did not observe any substantial entering of arterial flow into the aneurysm, nor could vortex flow be identified. Only in a very narrow region around the mesh of the stent, the outflow of the MNP was slightly delayed as shown in Fig. 6 at *t* = 11 s. A short time interval later, all the MNP had disappeared.

### Quantification

**Fig. 7:**
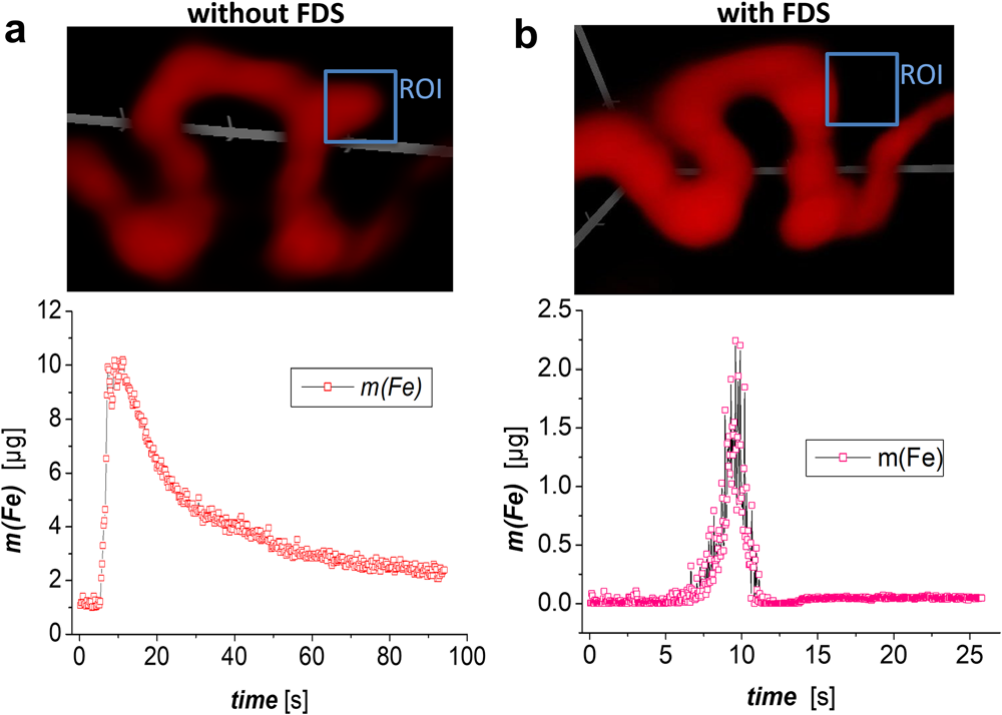
Quantification of MNP by using a region of interest (ROI) enclosing the aneurysm by MPI. In (**a**), quantified content of MNP over time without FDS is plotted with ROI position on top and (**b**) depicts the quantification with FDS.

The temporal evolution of the MNP content in the aneurysm, as illustrated in Fig. 7, facilitates the deviation of the flow into and out of the aneurysm. At the beginning we observe a large flow entry at *t* = 6 s in case of an aneurysm without FDS. This is followed by a long exponential decay. In the case of FDS, the flow entry is considerably lower with a notably lower magnitude, and the brief entry commences at *t* = 8 s, persisting for approximately 4 s. This is in contrast to the aneurysm without FDS, where the duration extends to over 100 s.

### Limitations

The present study constitutes a qualitative feasibility experiment, the objective of which is to demonstrate the capability of MPI to resolve flow patterns and detect flow modifications caused by an FDS. This experiment is distinct from a physiologically accurate reproduction of intracranial blood flow. Furthermore, the recognition of flow changes and turbulences directly in the MPI data necessitates specialized knowledge and experience. However, as demonstrated in refs. ^15,16^, it is possible to derive vectorial velocity data from the MNP distribution data, thereby facilitating the recognition process. In recent years, there has been a notable increase in the development of human MPI scanners^6,17,18^. However, at present, it remains challenging to predict whether and when their temporal and spatial resolution will be adequate to directly ascertain hemodynamic flow within the human body with sufficient precision. A further challenge for the transfer of this method is the question of how to realize an in vivo, homogeneous bolus formation of the tracer in the artery for the aneurysm.

### Application scenarios

Despite the diagnostic benefit of MPI flow imaging, which is certainly a long-term perspective due to the present lack of clinical, human-sized MPI scanners with sufficient spatial and temporal sensitivity, the application of MPI to assess the success of practicing endovascular surgical skills seems directly feasible even with the present preclinical MPI scanners. So, we see the following application scenarios:

#### Success verification of surgical training

**Fig. 8:**
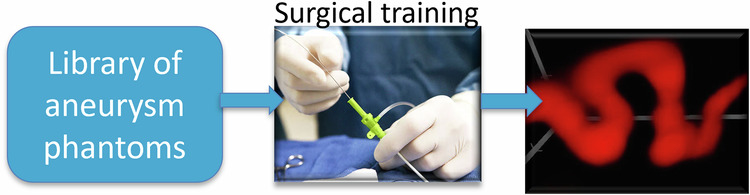
MPI to monitor stent insertion success for training surgical praxis. An aneurysm phantom will be selected from an existing digital library and printed. Next, the operator will train the insertion of the FDS into the phantom. Finally, success will be verified by imaging the flow changes with MPI.

There are several simulation models^19^ for training endovascular skills, but they all lack the ability of success verification^20^, i.e., how well the desired treatment goal is achieved, and to analyze errors if necessary. Here, MPI can directly assist by imaging of changes due to the surgical intervention with sufficient temporal and spatial resolution, see Fig. 8.

#### Patient-specific selection and adaptation of FDS before surgery

**Fig. 9:**
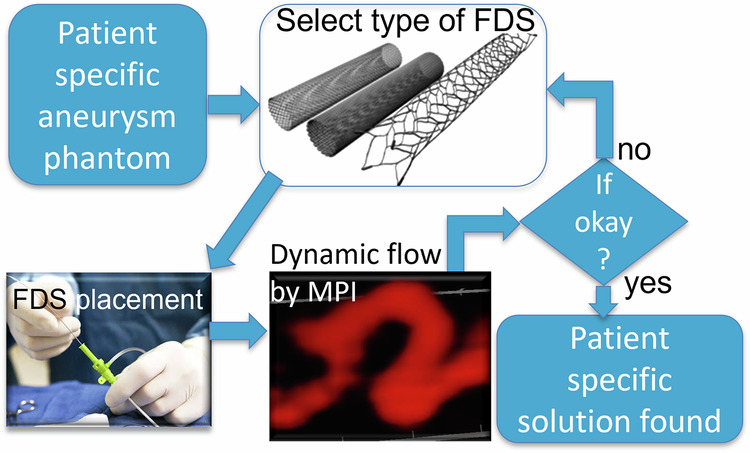
MPI to select a suitable stent containing patient specific aneurysm information for personalized FDS design. First, the aneurysm phantom is created from the patient's CT data set. Next, the FDS type is selected, along with the method by which the FDS will be adapted to the aneurysm, and the FDS is placed in the phantom. The success of the chosen FDS type and adaptation method is then evaluated using MPI flow analysis. If sufficient, a patient-specific solution is found. Otherwise, repeat with a different FDS type and/or adaptation method.

Furthermore, together with 3D printing of complex patient-specific aneurysms, MPI flow imaging could be another area of application for selecting a suitable personalized FDS^21,22^ and its individual adaptation, as shown in Fig. 9.

Here, the first step would be to create an aneurysm phantom from the patient’s CT aneurysm dataset and then select the FDS. The next step is to adapt the stent to the aneurysm. The MPI flow analysis then determines whether the flow is directed in such a way that the flow in the aneurysm becomes stagnant. If this is not the case, a new run with a different FDS type and/or a different adaptation must be performed until the patient-specific solution is achieved. This second scenario includes the optimization of two elements by the MPI feedback, the way of placement, and the type-selection of the FDS.

#### Development and optimization of new FDS designs

**Fig. 10:**
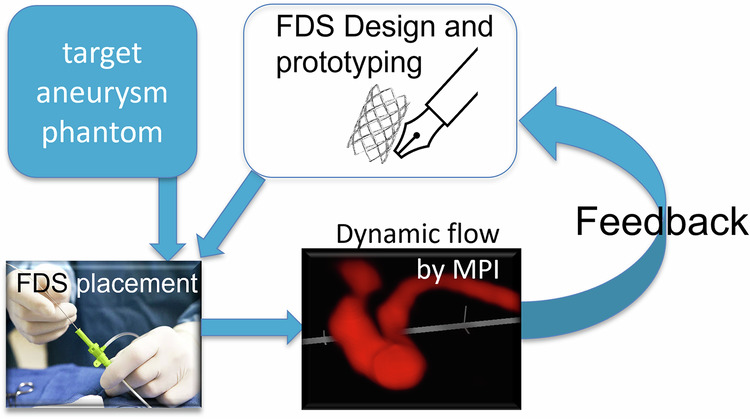
MPI flow analysis aided FDS design. An FDS is designed and inserted into a phantom of a target aneurysm through FDS placement. Our dynamic flow analysis generates detailed feedback on which part of the FDS should be redesigned based on regional flow.

This procedure would be analogous to the previous application example, yet it necessitates more detailed feedback see Fig. 10 regarding which component of the stent should be redesigned and the manner in which it should be modified, based on regional flow conditions as determined by MPI. In addition to the analysis of different flow conditions, the analysis of flow conditions for rupturing aneurysms could be expanded through the use of appropriate phantoms. Furthermore, the stents could be investigated regarding these conditions.

### Ethics statement

The authors declare that they have no known competing financial interests or personal relationships that could have appeared to influence the work reported in this paper. Ethical approval was not required for this study because only fully anonymized imaging data were used for 3D model reconstruction, and no identifiable patient information or direct patient involvement was included. Informed consent was waived due to the retrospective use of fully anonymized data.

## Conclusions

Based on the long residence time of the MNP in the aneurysm without an FDS resolved by MPI, we conclude that a stable vortex flow was formed in the aneurysm with little fluid exchange with the artery. Furthermore, we observed some vortex flows at strong curvatures in the artery (see Fig. 5), but these became only visible for a short time due to the higher fluid exchange rate at these locations. However, the sensitivity of MPI for the detection of such small flow turbulence is already evident. We therefore suppose that it will be possible to visualize by MPI the complex fluid mechanics of a wide-neck aneurysm and other variations, as well. The detection of the small flow at the mesh of the FDS (see Fig. 6, *t* = 5 s) already demonstrates the high performance of the MPI and opens the possibility of achieving even more complete suppression of the flow in the aneurysm by using a different stent variant with, for example, narrower meshes. In total, already here the powerful application of MPI to visualize many details of the fluid mechanics in an aneurysm has been successfully demonstrated. In addition to the potential use of this imaging as feedback for training in endovascular surgical skills and the individualization of flow diverter solutions for personalized treatment of complex aneurysms, there is another application scenario where this feedback can be used to optimize stent designs for different endovascular treatments.

We will continue our work with other more complex aneurysms like fussy-form or wide-neck aneurysms and with a more detailed setup for several realistic flow conditions, like systolic and diastolic flow rates and improved phantoms. Furthermore, the analysis will be expanded through the derivation of vectorial velocity data from MPI distribution data, as previously outlined in refs. ^15,16^, with the objective of enhancing the detection of flow and flow alterations.

## Supplementary information

Transparent Peer Review file

Reporting summary

## Data Availability

The data that support the findings of this study are available from the authors on reasonable request; see “Author contributions” for specific data sets.
