# Supplementary material for: Millisecond magnetic particle imaging of a cerebral aneurysm phantom with and without flow diverter stent
Source: Commun Eng. 2026 Jul 29;5:139. doi: 10.1038/s44172-026-00726-0 (PMC13421539; doi:10.1038/s44172-026-00726-0)
Supplement: Supplementary file 1 — Transparent Peer Review file [file 44172_2026_726_MOESM1_ESM.pdf]

nature portfolio

Peer Review File

Millisecond Magnetic Particle Imaging of a cerebral aneurysm phantom with and without flow diverter stent

Corresponding Author: Dr Olaf Kosch

This file contains all reviewer reports in order by version, followed by all author rebuttals in order by version.

Version 0:

Reviewer comments:

Reviewer #2

(Remarks to the Author)
Dear authors,

thank you for your mansucript. You adress an interesting topic as especially cerebral imaging is very promising to be performed with MPI. Unfortunately, your experiments are limited to a single aneurysm morphology and the results are soley described qualitatively. Furthermore, the discussion of your findings is very short and you do not adress any limitations of this proof-of-concept study. I made some comments in the attached pdf which hopefully help to improve your paper.

best regards
Your reviewer

Reviewer #3

(Remarks to the Author)
comments to the authors
Review of "Millisecond Magnetic Particle Imaging of a cerebral aneurysm phantom with and without flow diverter stent"
The authors present a study using Magnetic Particle Imaging (MPI) to visualize flow dynamics in a patient-specific aneurysm phantom, both untreated and treated with a PED. They demonstrate prolonged tracer retention and vortex flow in the untreated case and near-complete flow suppression into the aneurysm when a PED is applied. They further propose applications in surgical training, personalization of FDS selection, and device development.

The manuscript is interesting and a relevant step for the MPI community. The experiments show the promise of MPI for qualitative flow assessment.
However, several methodological aspects lack detail, multiple interpretations appear overstated, and central claims (millisecond resolution, vortex detection, physiological realism) require substantial clarification.

comments:
1. Description of the 3D printing and extraction/post-processing workflow
The phantom manufacturing section (II.I) provides a general outline ("washed with isopropanol… cleaned… cured at 80°C"), but the extraction process itself of the data of the inner lumen structure is not described in sufficient technical detail.
Since the extraction this 3D information out of patient data can be quite difficult, the authors should provide more details about this step.
In addition, was there any coating applied on the inner vessel walls of the 3D-printed phantom in a post-processing step to approximate realistic vascular compliance or friction?
Furthermore, can you provide a statement about the used material ("elastic 50A resin") regarding its mechanical and tribological comparison to endothelium?
2. Physiological relevance of flow rate and viscosity
The authors use a constant flow of 3 mL/min (II.II) with a glycerin/water mixture intended to mimic viscosity. However, ICA flow in humans is typically 200–350 mL/min, with significant pulsatility. The authors do not provide a justification for why such a low flow rate was chosen beyond "to detect late vortex flow" (III.I), nor do they discuss how this affects physiological relevance.

Discuss explicitly whether the chosen flow rate and viscosity produce Reynolds numbers comparable to human cerebral arteries. If not, clearly state that the experiment evaluates qualitative feasibility only, not physiological accuracy.
3. Air-bubble prevention
Did the authors require a bubble trap in their setup or is there no issue expected with such things?

4. “Millisecond” imaging
“Thus, in case of a 3D acquisition scheme one total Lissajous-trajectory is completed after a period of 21.5 ms, allowing the reconstruction of a full 3D dataset of the FOV with millisecond resolution“
One full acquisition can be performed in 21.5ms, which represents one full volume. What the authors mean with ‘millisecond’ temporal resolution? The maximum number of volumes per second is 1000/21.5ms=46 volumes per second. when talking about milliseconds temporal resolution, I understand something different!? Or did you perform a ‘sliding’ window reconstruction. When doing so, please provide more information for such a reconstruction step. When not, then please think about the title of this manuscript.
5. Spatial resolution
The authors state a spatial resolution of about 3.7mm. Following the rule of thumb for MPI systems I would expect a spatial resolution of about 2-3 mm?
Furthermore, the aneurysm dimension is ~4-6 mm: is the reached spatial resolution high enough?
6. “flow properties” and “vortex flow”
I would be careful with such a statement: “MNP used as probes were capable to resolve the flow properties in the region of the aneurysm and the action of diverter stent in the aneurysm.” since the MPI cannot provide specific flow information such as MRII with vectorial flow or something
figure 3+4: what does the author mean with “vortex flow”: MPI ‘just’ provides the distribution of MNPs within the vessel structure with a ‘high’ temporal resolution with no more information are visible in the picture?
I know what the authors mean, but I think it is not directly possible to see that in the MPI data?!
Maybe by showing that with an established imaging technique could strengthen this statement.

minor comments
-maybe it is not useful to provide this information: “installed at Charité University Hospital Berlin”

-discussion: could you provide a more detailed discussion regarding transferring this approach to human-scale.
are there limitations for this approach, etc.

-is it is possible to provide quantitative information out of the reconstructed data? If so, maybe some colorbars could be added to the figures.

Version 1:

Reviewer comments:

Reviewer #2

(Remarks to the Author)
Dear authors,
thank you for the revised paper. You really improved your work and I only have a single minor issue left:

Fig.7 with Flow diverter:
It would be helpful to display a larger range on the x-axis so that the reader can see what happens after the initial peak. In addition, the corresponding text should address the fact that the signal intensity of the peak is significantly lower with the flow diverter than without it. In the current version, the text sounds as if the flow diverter only changes the timing of the inflow into the aneurysm, rather than also reducing its magnitude.

Best regards
Your reviewer

Open Access This Peer Review File is licensed under a Creative Commons Attribution 4.0 International License, which permits use, sharing, adaptation, distribution and reproduction in any medium or format, as long as you give appropriate credit to the original author(s) and the source, provide a link to the Creative Commons license, and indicate if changes were made.
In cases where reviewers are anonymous, credit should be given to 'Anonymous Referee' and the source.
The images or other third party material in this Peer Review File are included in the article's Creative Commons license, unless indicated otherwise in a credit line to the material. If material is not included in the article's Creative Commons license and your intended use is not permitted by statutory regulation or exceeds the permitted use, you will need to obtain permission directly from the copyright holder.
To view a copy of this license, visit https://creativecommons.org/licenses/by/4.0/

Millisecond Magnetic Particle Imaging of a cerebral aneurysm phantom with and without flow diverter stent

Manuscript Number: COMMSENG-25-0752-T

We thank all reviewers for their positive, constructive feedback and the helpful comments to our manuscript. We addressed all comments highlighted by the reviewers in the following way: Original comments of reviewers in black, authors' answers in blue, changes to the manuscript highlighted in yellow in the attached document.

Reviewer #2:

Dear authors,

thank you for your manuscript. You address an interesting topic as especially cerebral imaging is very promising to be performed with MPI. Unfortunately, your experiments are limited to a single aneurysm morphology and the results are soley described qualitatively. Furthermore, the discussion of your findings is very short and you do not adress any limitations of this proof-of-concept study. I made some comments in the attached pdf which hopefully help to improve your paper.

best regards
Your reviewer

Why do you highlight the frame duration here? To achieve a single image in miliseconds is very typical for MPI...

We highlight this here, because in our opinion the high temporal resolution of fast MPI (calibration-based MPI using three drive fields, traveling wave MPI) provides sufficient high temporal resolution to enable flow detection.

To which aspect the 80% are related?

We thank the reviewer for this important question. Indeed, the number of cases varies depending on specific parameters of the aneurysms (localization, size, etc.). We therefore changed the sentence to: "Undetected, asymptomatic brain aneurysms are associated with a high risk of progressive expansion, followed by rupture and hemorrhage, which in turn results in a high risk of mortality and morbidity."

"Can close" is very unprecise. Please name the fact that a thrombosis happens which causes an occlusion of the aneurysm here.

We thank the reviewer for this comment and have clarified this by adding the sentence: "The stagnation of blood flow leads to the formation of a thrombus within the aneurysm, resulting in its occlusion."

What exactly is the link between this fact and your study? What is the gap of knowledge and your hypothesis?

We thank the reviewer for these questions. We have added a few sentences to clarify the knowledge gap and our hypothesis. “One of the main reasons for this high complexity is the lack of comprehensive hemodynamic data on the flow inside the aneurysm and the artery when fitting the FDS. In this study, we want to clarify whether MPI is sensitive enough to provide such detailed hemodynamic data and can help reducing the complexity of FDS adaptation. With this in mind, we analyzed the effect of a flow diverter stent placed in an aneurysm phantom by preclinical MPI on the hemodynamic flow conditions compared to the untreated aneurysm situation.”

“Based on MRI-data sets of a patient-specific internal carotid artery (ICA) aneurysm, two identical aneurysms phantoms were made at University Hospital LMU Munich by additive manufacturing.” Please name the specific vessel section hier.

We thank the reviewer for this comment. We have added the vessel section, and we have to apologize to have mentioned MRI for extracting the anatomical structures since it was a CT based data set. “Two identical aneurysm phantoms were fabricated by additive manufacturing at the University Hospital of the LMU in Munich, Germany, based on CT data of a patient-specific internal carotid artery (ICA) aneurysm in the ophthalmic portion.”

Figure 1, The aneurysm is not clearly visible. Please use an arrow to indicate the aneurysm.

We thank the reviewer for this request. The aneurysm itself is hidden by the supply structures and not visible and we therefore added this information.

“Figure 1: Cerebral artery phantom directly after printing (the printing support structure is still visible, the aneurysm itself is hidden by the support structure) by stereolithography with silicon-like resin.”

Pulsatile or continous flow?

We thank the reviewer for this comment. We used in this study continuous flow and have added this information to the manuscript.

Was really the whole phantom covered by the FOV?

We thank the reviewer for this question and clarified it in the sentence: “We chose these gradient and drive fields to obtain a sufficiently large physical FOV with a size of 40 x 40 x 20 mm^3 to cover the arterial phantom without the inlet and the outlet sections.”

the discussion part is very short. a limitations section is totally missing...

We thank the reviewer for pointing out the lack of discussion related to limitations. Thus, we extended this section and added section III.IV. Limitations.

This whole section only describes the particle signal qualitatively. There is no quantitative analysis e.g. the signal intensity course inside the aneurysm vs. outside...

We apologize for this lack of quantitative information. To clarify, we imaged pictures by using the whole 3D-data for one timestep or the whole 4D-data in the video sequences and no single 2D cross-sections, with the objective of identifying all pertinent details, but this works not as quantitative imaging. We added this information to section II.III. "The utilization of MIPAV entails the rendering of the data set as a 4D sequence, thereby facilitating the visualization of the entire dataset, as opposed to merely individual cross-sections, with the objective of identifying all pertinent details. For quantitative analysis, the 4D-data were read into MATLAB (MathWorks, Inc.), where a region of interest (ROI) close to the aneurysm was defined with a size of 7.5 x 7.5 x 4.5 mm^3 for both cases (without and with FDS). The content of MNP within the ROI was plotted over time to visualize the amount of flow entering the aneurysm and the retention of MNP within the aneurysm."

This whole section only describes the particle signal qualitatively. There is no quantitative analysis e.g. the signal intensity course inside the aneurysm vs. outside...

We fully agree with the reviewer. Therefore, we added a section III.III Quantification for quantitative results.

„III.III. Quantification

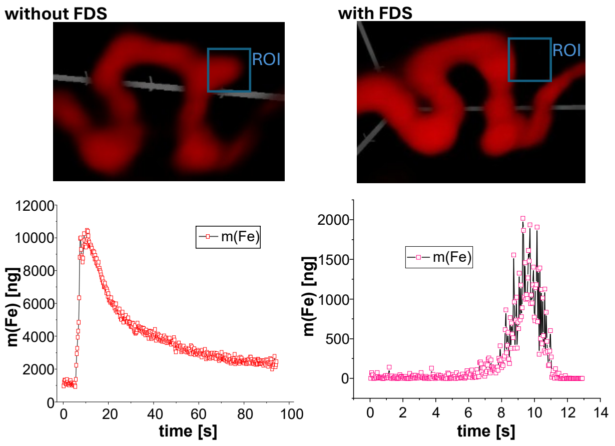

Figure 7: Content of MNP in the aneurysm, Left: without FDS and Right: with FDS, Top: position of the ROI close to the aneurysm, Bottom: quantified content of MNP over time

The temporal evolution of the MNP content in the aneurysm, as illustrated in Fig. 7, facilitates the deviation of the flow into and out of the aneurysm. At the beginning we observe a large flow entry at *t* = 6 s in case of an aneurysm without FDS. This is followed by a long exponential decay. In the case of FDS, a brief entry commences at t = 8 s, persisting for approximately 4 s. This is in contrast to the aneurysm without FDS, where the duration extends to over 100 s."

“In areas of strong curvature and widening of the artery, such as p1 and p2, we also observe a weak vortex formation, but much more transient than in the aneurysm.” I doubt that you can be sure to call it a vortex, as you have only a delayed signal and no morphological flow information.

We thank the reviewer for this comment, and we have changed “observe” to “assume”. Acknowledging your feedback, we understand that it is mandatory to examine the 4D data sequence rather than a static image. We added a link for accessing the video-sequences and added some text in section III.:

“The subsequent images depict several of the *key* scenes from 4D data; nevertheless, the videos offer a more exhaustive illustration of the hemodynamic flow. Video sequences are accessible under the following link (press download and open it in MS *PowerPoint or OpenOffice*): https://box.ptb.de/getlink/fiHDZn8LwhBTwejPCwZE86e4/aneurysm_videos.ppsx”

“Due to the limitation of the static image, we comment to look at the first video sequence in the MS PowerPoint-file. In areas of strong curvature and widening of the artery, such as p1 and p2, we also assume a weak vortex formation, but much more transient than in the aneurysm.”

“In the aneurysm phantom with flow diverter stent, the MPI reconstructions reveal that the MNP much faster pass the aneurysm region, as if there was no vortex flow to observe in the aneurysm.” Can you give a factor?

We thank the reviewer for pointing out this interesting feature. To address the duration of the passage we clarified in the new added section III.III Quantification. “In case of FDS, a brief entry commences at t = 8 s, persisting for approximately 4 s. This is in contrast to the aneurysm without FDS, where the duration extends to over 100 s.”

Reviewer #3:

comments to the authors
Review of “Millisecond Magnetic Particle Imaging of a cerebral aneurysm phantom with and without flow diverter stent”
The authors present a study using Magnetic Particle Imaging (MPI) to visualize flow dynamics in a patient-specific aneurysm phantom, both untreated and treated with a PED. They demonstrate prolonged tracer retention and vortex flow in the untreated case and near-complete flow suppression into the aneurysm when a PED is applied. They further propose applications in surgical training, personalization of FDS selection, and device development.

The manuscript is interesting and a relevant step for the MPI community. The experiments show the promise of MPI for qualitative flow assessment.
However, several methodological aspects lack detail, multiple interpretations appear overstated, and central claims (millisecond resolution, vortex detection, physiological realism) require substantial clarification.

comments:
1. Description of the 3D printing and extraction/post-processing workflow
The phantom manufacturing section (II.I) provides a general outline ("washed with isopropanol... cleaned... cured at 80°C"), but the extraction process itself of the data of the inner lumen structure is not described in sufficient technical detail.
Since the extraction this 3D information out of patient data can be quite difficult, the authors should provide more details about this step.
In addition, was there any coating applied on the inner vessel walls of the 3D-printed phantom in a post-processing step to approximate realistic vascular compliance or friction?

We thank the reviewer for these important comments and requests. We apologize that the original manuscript did not describe the inner lumen extraction and reconstruction workflow in sufficient technical detail, although this is a crucial step for generating patient-specific phantoms. We therefore expanded Section II.I to explicitly document the complete pipeline from medical imaging to STL generation.
In the revised manuscript, we now specified that the CT datasets were acquired using an Integris Allura FD20 system (Philips Healthcare) and reconstructed on an Xtravision Philips workstation within a 256 × 256 × 256 matrix. The images were exported in DICOM format and imported into OsiriX v5.6, where the ROI was defined and a dedicated preprocessing ("reparation") step was performed, including artifact deletion, segmentation correction, and smoothing of the ROI. The aneurysm lumen was then segmented and iteratively refined to obtain a clean lumen volume, which was subsequently reconstructed into a triangulated 3D surface mesh and exported as an STL file for downstream volume grid generation and phantom fabrication.

"The patient-specific aneurysm lumen geometry was reconstructed from contrast-enhanced CT imaging datasets acquired using an Integris Allura FD20 system (Philips Healthcare, Best, The Netherlands). The image volumes were reconstructed on an Xtravision Philips workstation within a 256 × 256 × 256 matrix and exported in DICOM format for further processing. The DICOM datasets were imported into OsiriX v5.6 (Pixmeo SARL, Bernex, Switzerland), where the region of interest (ROI) containing the aneurysm and adjacent parent vessels was selected. A preprocessing ("reparation") step was performed to improve segmentation fidelity, including removal of acquisition-related artifacts, correction of discontinuities or spurious structures, and smoothing of the ROI to ensure a consistent representation of the inner lumen surface.
Following this preparation stage, the aneurysm lumen was segmented in OsiriX using intensity-based and semi-automatic tools, allowing separation of the contrast-enhanced blood pool from surrounding tissue. The segmentation results were iteratively refined to

obtain a clean and continuous lumen volume. The final lumen segmentation was then converted into a triangulated 3D surface model, and the reconstructed geometry was exported in STL format. This STL model served as input for subsequent processing steps, including volume grid generation, phantom manufacturing, and computational analyses."

No, there no coating has been applied, since we used a glycerol/water mixture.

Furthermore, can you provide a statement about the used material ("elastic 50A resin") regarding its mechanical and tribological comparison to endothelium?

Elastic 50A Resin V2 vs endothelium (endothelial cells)

Stiffness (Young's modulus) — the key mismatch

- Endothelial cells: commonly reported ~0.2–7 kPa depending on method, cell state, and location.
- The resin (Shore A 55A): a common durometer→modulus conversion gives ~1.9 MPa (≈ 1900 kPa).

Order-of-magnitude comparison:
1.9 MPa / (0.2–7 kPa) ≈ ~270× to ~9500× stiffer than endothelial cells.

Thickness scale is totally different

- Cerebral endothelial membrane thickness: reported around 0.3–0.5 μm (sub-micron).
  Your printed resin wall is typically hundreds of microns to millimeters.

Strength / elongation numbers don't map well

Your tensile strength and elongation (e.g., 3.4 MPa, 160%) describe a bulk polymer. Endothelium is a living, active, viscoelastic cell layer whose "failure" is usually discussed in terms of junction integrity, detachment, permeability, etc., not bulk tensile rupture.

What this means practically

- If your goal is hemodynamics in the lumen: endothelium mechanics usually doesn't need to be matched; the endothelial layer is too thin to affect lumen shape at the macroscopic scale.
- If your goal is biomimetic wall compliance or cell-interaction experiments: Elastic 50A is far too stiff as an "endothelium analog."

Our goal is hemodynamics in the lumen.

2. Physiological relevance of flow rate and viscosity

The authors use a constant flow of 3 mL/min (II.II) with a glycerin/water mixture intended to mimic viscosity. However, ICA flow in humans is typically 200–350 mL/min, with significant pulsatility. The authors do not provide a justification for why such a low flow rate was chosen beyond "to detect late vortex flow" (III.I), nor do they discuss how this affects physiological relevance.
Discuss explicitly whether the chosen flow rate and viscosity produce Reynolds numbers comparable to human cerebral arteries. If not, clearly state that the experiment evaluates qualitative feasibility only, not physiological accuracy.

We thank the reviewer for this important comment. We agree that using a constant flow rate of 3 mL/min does not represent physiological human ICA flow rates (~200–350 mL/min) and does not capture the pulsatile character of intracranial circulation. In our study, the primary objective of selecting this low, steady flow was not to reproduce physiological boundary conditions but to enable robust visualization of intra-aneurysmal flow behavior with millisecond MPI, particularly the detection of long-residence-time vortex patterns ("late vortex flow"), which require extended observation periods and stable tracer detectability.

As described in Section II.II, we used a glycerol/water mixture (59.1% glycerol, 40.9% ddH_2O) to mimic the viscosity of blood while maintaining a controlled experimental setup using a syringe pump. However, due to the substantially reduced flow rate, the resulting Reynolds numbers are expected to be lower than those in physiological ICA conditions, meaning that the experiment is not intended to provide quantitatively physiological hemodynamic conditions.

To include these points, we revised the manuscript to explicitly state that the chosen flow condition represents a qualitative feasibility experiment designed to demonstrate the capability of MPI to resolve flow patterns and detect flow modifications caused by a flow diverter stent, rather than a physiologically accurate reproduction of intracranial blood flow. We also added a clear limitation statement and outlook that future work will include more realistic flow conditions, including systolic/diastolic pulsatile regimes, as already outlined in the conclusions of the manuscript.

" The selection of this flowrate was made to illustrate the capacity of MPI to discern even minor turbulences. The dependence of the intensity of turbulences and vortices on the arterial flow rate is shown for untreated and treated aneurysms in [10]."

3. Air-bubble prevention
Did the authors require a bubble trap in their setup or is there no issue expected with such things?

We thank the reviewer for the comment. We have not used a bubble trap and cleaned the system from air bubbles by pumping the fluid through the system. We have added a sentence in section II.II.: "The removal of air bubbles was accomplished by pumping the mixture through the tubes and the phantom." A bubble trap may change the bolus

formation.

4. “Millisecond” imaging
“Thus, in case of a 3D acquisition scheme one total Lissajous-trajectory is completed after a period of 21.5 ms, allowing the reconstruction of a full 3D dataset of the FOV with millisecond resolution“
One full acquisition can be performed in 21.5ms, which represents one full volume. What the authors mean with ‘millisecond’ temporal resolution? The maximum number of volumes per second is 1000/21.5ms=46 volumes per second. when talking about milliseconds temporal resolution, I understand something different!? Or did you perform a ‘sliding’ window reconstruction. When doing so, please provide more information for such a reconstruction step. When not, then please think about the title of this manuscript.

We apologize but we can not follow the argumentation of the reviewer here, because a 3D image every ~ 20 ms is in the millisecond range. Since we have a full 3D image providing the spatial resolution, so we do not have to use any sliding window.

5. Spatial resolution
The authors state a spatial resolution of about 3.7mm. Following the rule of thumb for MPI systems I would expect a spatial resolution of about 2-3 mm?
Furthermore, the aneurysm dimension is ~4-6 mm: is the reached spatial resolution high enough?

We thank the reviewer for this comment. The principle of thumb may not be clearly defined. We estimated the spatial resolution by using the full-width-at-half-maximum criterion. This means that we can distinguish between two areas if the amplitude is less than 50% of the maximum between these two regions. But of course, the two regions can also be separated using e.g. a 70% criterion. It is important to note that a resolution of 3.7 mm does not preclude the ability to discern objects that are smaller than 3.7 mm. Additionally, the resolution is enhanced in the *z*-direction due to the higher gradient. Increasing resolution is generally possible by using a higher selection field, which in turn allows sharper distributions to be achieved. However, it should be noted that the field of view (FOV) will be smaller. In our investigation we aimed for a FOV that contains most of our aneurysm phantom resulting in a slightly reduced spatial resolution.

“The selection of this flowrate was made to illustrate the capacity of MPI to discern even minor turbulences. The dependence of the intensity of turbulences and vortices on the arterial flow rate is shown for untreated and treated aneurysms in [10].“

6. “flow properties” and “vortex flow”
I would be careful with such a statement: “MNP used as probes were capable to resolve

the flow properties in the region of the aneurysm and the action of diverter stent in the aneurysm." since the MPI cannot provide specific flow information such as MRII with vectorial flow or something
figure 3+4: what does the author mean with "vortex flow": MPI 'just' provides the distribution of MNPs within the vessel structure with a 'high' temporal resolution with no more information are visible in the picture?
I know what the authors mean, but I think it is not directly possible to see that in the MPI data?!
Maybe by showing that with an established imaging technique could strengthen this statement.
We thank the reviewer for this feedback and fully agree that the recognition of flow properties like vortex flow require some experience and knowledge of such flow simulation data and surely, it would be easier to look at temporal data, the 3D data sets compiled into video sequences. We therefore added a link for these video sequences. It has been observed that there is a flow entry to the lower part of the aneurysm. Over time, this entry moves to the upper part of the aneurysm (against the direction of the main flow in the artery), where a small backflow commences at the upper edge of the aneurysm, returning to the artery. It is our professional opinion that these movements can only be explained by the rotational movement of a vortex flow.

It is evident that we record by MPI the path of the MNP over time, from which vectorial velocity data can be derived as has been shown in [15, 16]. In our future work we will also derive vectorial flow data from the 4D-MPI data for our method to make the recognition of flow turbulences easier, especially for static pictures. We added this information to the manuscript.

"Furthermore, the analysis will be expanded through the derivation of vectorial velocity data from MPI distribution data, as previously outlined in [15], [16], with the objective of enhancing the detection of flow and flow alterations."

minor comments
-maybe it is not useful to provide this information: "installed at Charité University Hospital Berlin"

We wonder about this request of the reviewer and would like to know why it is not useful to assign the specific MPI device that has been used for our investigations. Though the Bruker MPI scanners are as commercial products of be assumed to be identical there are features that distinguish MPI scanner sites from others. As an example we mention the existence of different separate receive coils.

-discussion: could you provide a more detailed discussion regarding transferring this approach to human-scale.
are there limitations for this approach, etc.

We thank the reviewer for addressing this. In the added section **III.IV. Limitations** where the limitations of MPI in the context of this article have been discussed we additionally included in this section a paragraph addressing the limitations of a transfer to human scale applications.

“In recent years, there has been a notable increase in the development of human MPI scanners [6], [17], [18]. However, at present, it remains challenging to predict whether and when their temporal and spatial resolution will be adequate to directly ascertain hemodynamic flow within the human body with sufficient precision. A further challenge for the transfer of this method is the question of how to realize an in vivo, homogeneous bolus formation of the tracer in the artery for the aneurysm.”

-is it is possible to provide quantitative information out of the reconstructed data? If so, maybe some colorbars could be added to the figures.

We thank the reviewer for also requesting quantitative information. The pictures of the MNP distributions were made by using the whole 3D-data for one timestep or the whole 4D-data in the video sequences and no single 2D cross-sections, with the objective of identifying all pertinent details, but this works not as a quantitative imaging. Therefore, we added this information to section II.III of the manuscript: “The utilization of MIPAV entails the rendering of the data set as a 4D sequence, thereby facilitating the visualization of the entire dataset, as opposed to merely individual cross-sections, with the objective of identifying all pertinent details.”

Attachment: Manuscript_yellow_marked_changes_for_reviewers.pdf

Best regards,

the authors.

Millisecond Magnetic Particle Imaging of a cerebral aneurysm phantom with and without flow diverter stent

Manuscript Number: COMMSENG-25-0752-T

We thank all reviewers for their positive, constructive feedback and the helpful comments to our manuscript. We addressed all comments highlighted by the reviewers in the following way: Original comments of reviewers in black, authors' answers in blue, changes to the manuscript highlighted in yellow in the attached document.

<u>Reviewer #2 (Remarks to the Author):</u>

Dear authors,
thank you for the revised paper. You really improved your work and I only have a single minor issue left:

Fig.7 with Flow diverter:
It would be helpful to display a larger range on the x-axis so that the reader can see what happens after the initial peak. In addition, the corresponding text should address the fact that the signal intensity of the peak is significantly lower with the flow diverter than without it. In the current version, the text sounds as if the flow diverter only changes the timing of the inflow into the aneurysm, rather than also reducing its magnitude.

Best regards
Your reviewer

We thank the reviewer for this comment and have clarified the corresponding text:

"In the case of FDS, the flow entry is significantly lower with a notably lower magnitude, and the brief entry commences at t = 8 s, persisting for approximately 4 s."

In Figure 7, we have extended the range after the initial peak in the case of FDS. We have also changed the units to µg for ***m(Fe)*** to make the numbers easier to read.

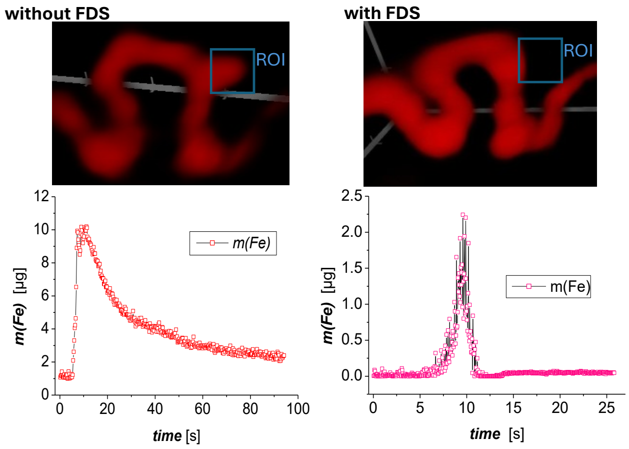

Figure 7: Content of MNP in the aneurysm, Left: without FDS and Right: with FDS, Top: position of the ROI close to the

aneurysm, Bottom: quantified content of MNP over time.

Attachment: Manuscript_yellow_marked_changes_for_reviewers2.pdf

Best regards,

the authors.

Millisecond Magnetic Particle Imaging of a cerebral aneurysm phantom with and without flow diverter stent

Abstract:

Undetected, asymptomatic brain aneurysms include the high risk of progressive expansion, followed by rupture, hemorrhage and finally death in approximately 80% of the cases. Therefore, imaging techniques to assess the state of aneurysms and the success of the treatment of aneurysms are of high relevance. For this purpose, we manufactured artery phantoms with aneurysms by 3D silicon printing. Using MRI data sets including cerebral aneurysms that were specific to patients, the artery was created. By Magnetic Particle Imaging (MPI), we were able to resolve the location and movement of the tracer in the different parts of the artery phantom with millisecond resolution. To assess the success of treating aneurysms with flow diverter stents, we compared flow MPI measurements in a 3D-printed artery without a flow diverter stent with images in a treated artery with a flow diverter stent inside the vessel. Our results provide valuable information on the capabilities and prospects of MPI of vascular structures and the detection and quantification of flow changes in untreated and treated aneurysms for future in vivo applications.

I. Introduction

Cerebral aneurysm is a disease characterized by local dilatation of the arterial walls in the intracranial vasculature, usually at arterial curves and bifurcations in the circle of Willis [1]. Such pathological weakness of an artery carries a high risk of progressive dilation with subsequent bursting or rupture of the artery leading to a subarachnoid hemorrhage, which is associated with high mortality and morbidity [2]. Understanding the hemodynamic mechanisms involved is critical to identifying effective treatment options to reduce the risk of rupture and bleeding in cerebral aneurysms. An effective endovascular treatment is the use of a flow diverter stent [3],[4] to divert blood flow away from the aneurysm. Ideally, the blood flow in the aneurysm is stopped and the aneurysm can close. In this paper, we show the capability of Magnetic Particle Imaging (MPI) to detail the blood flow in an aneurysm and its changes through the presence of a flow diverter stent. With its high temporal and spatial resolution in detecting magnetic nanoparticles (MNP) employed as a tracer in a vascular system, MPI offers great potential for the analysis of hemodynamic situation of aneurysms. The capability of MPI for flow imaging was demonstrated recently in an internal carotid artery phantom with a realistic 3D aneurysm [5] and compared with magnetic resonance imaging and dynamic digital subtraction angiography, where it was possible to track a MNP bolus through the model and to estimate the average flow velocity by MPI. However, it is difficult to discern further details of the hemodynamic flow due to the noise present in these MPI results. Though, already a human brain MPI scanner [6] exists for stroke studies, it only has a reduced suitability for hemodynamic imaging due to its low spatial and especially, temporal resolution. The sensitivity and signal-to-noise ratio of our scanner have been enhanced by utilizing separate receiving coils and additional filters. The primary objective of this enhancement is to enable quantitative cell detection [7]. However, we also utilize this increased sensitivity for our hemodynamic imaging. The artefact-free MPI imaging of a straight vessel phantom to visualize the stent lumen in intracranial flow diverters was reported in [8].

Flow diverter stents (FDS) are often used to treat large or giant aneurysms and wide-neck and fusiform aneurysms, where stent adaptation is a complex task with an overall complication rate of 17% [9]. In ruptured aneurysms, the complication rate rises to 30.6%. With this in mind, we analyzed the effect of a flow diverter stent placed in an aneurysm phantom by preclinical MPI on the hemodynamic flow conditions compared to the untreated aneurysm situation. By this, we want to assess the capability of MPI for imaging hemodynamic flow and flow changes caused by endovascular intervention. For this purpose, we imaged here the dynamics of untreated and flow diverter stent treated patient-specific cerebral aneurysms to demonstrate the benefit of MPI for an endovascular treatment. To link high-resolution temporal and spatial MPI with patient data, we used 3D printing as a bridging technology.

II. Material and methods

II.I. Artery aneurysm phantom manufacturing

Based on MRI-data sets of a patient-specific internal carotid artery (ICA) aneurysm, two identical aneurysms phantoms were made at University Hospital LMU Munich by additive manufacturing. The aneurysm was of saccular morphology (4.5 mm dome height, 6 mm cross-section, 4 mm neck, 3 mm parent artery diameter). The MRI data sets were first processed to a printable model and produced on a stereolithography printer (Form 2, Formlabs GmbH, Germany) with a minimal layer height of 25 µm by use of silicon material (elastic 50A resin, FormLabs GmbH, Germany). Fig. 1 shows one phantom just after printing so

that the support structure still is visible. After that, the phantoms were first washed with isopropanol (Merck KGaA, Germany) using an ultrasonic bath and tempered at 50 °C, a cleaning step using a compressed-air gun followed. Finally, a post-cure procedure was carried out at 405 nm light wavelength and heating the phantoms at about 80 °C for 60 min. Then, the 3D printing supports were removed for better positioning during MPI measurements.

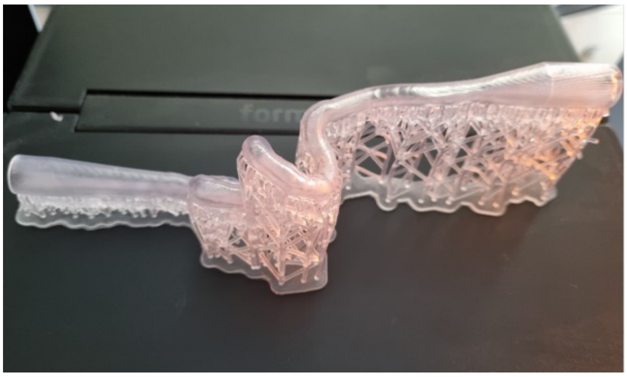

Figure 1: Cerebral artery phantom with aneurysm directly after printing (the printing support structure is still visible) by stereolithography with silicon-like resin.

Into one of the two phantoms a flow divertering stent Pipeline Embolization Device (PED; Medtronic Neurovascular, Irvine, California, USA was catheterized to the aneurysm location and fixated by dilation.

II.II. Measurement setup

The arterial phantom was placed on the animal bed and positioned inside the detection coils of the MPI scanner, see Fig. 2. The vascular system of the phantom was connected to a syringe pump (LA-800, Landgraf Laborsysteme HLL, GER) to provide different, defined flow rates. In preparation of the experiments, the syringe, tubing, and phantom were filled with a glycerine-water mixture (59.1% glycerin, 40.9% distilled deionised water (ddH2O)) to mimic the viscosity of blood. During the MPI data acquisition an MNP bolus of 100 µL Perimag (Micromod Partikeltechnologie, GER) at an iron concentration c(Fe) = 50 mmol/L was administered to the vascular phantom and pumped through it at flow rate of Q = 3 mL/min. The MNP bolus was prepared by placing it in a tube section outside the scanner.

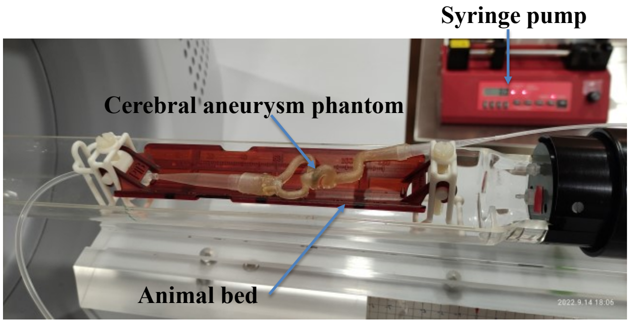

Figure 2: Vascular phantom connected to syringe pump prepared for the MPI measurements.

Prior to the initiation of the experiment, it was imperative to ensure that the bolus remained intact. Then afterwards, as the bolus passed through the cerebral artery and the aneurysm, the bolus was swirled and diluted in the medium by the passage.

II.III. MPI parameter

All MPI measurements were performed using a preclinical MPI system (Bruker MPI 25/20 FF) installed at Charité University Hospital Berlin, equipped with an additional separate receiving coil to increase the sensitivity [10] and additional receive filters (Bruker). This system is based on the movement of a field-free-point (FFP) through the whole imaging volume and employs the system function (SF) approach for image reconstruction [11]. A selection gradient field of ΔB = 1.2 T/m in the z-direction and ΔB = 0.6 T/m in x- and y-directions was used to generate the FFP and was implemented for all measurements presented in this work. By moving the FFP through the field of view (FOV), a spatially encoded signal of the whole imaging volume can be detected. The movement of the FFP on a closed 3D Lissajous-trajectory is accomplished by applying three orthogonal drive-fields oscillating at slightly different excitation frequencies at about 25 kHz (2.5 MHz divided by 102/96/99 in x-/y-/z-direction) with amplitudes of B = 12 mT. We chose these gradient and drive fields to obtain a sufficiently large physical FOV with a size of 40 x 40 x 20 mm^3 to cover the arterial phantom. Thus, in case of a 3D acquisition scheme one total Lissajous-trajectory is completed after a period of 21.5 ms, allowing the reconstruction of a full 3D dataset of the FOV with millisecond resolution. The system function (SF) required for image reconstruction was recorded with a point-like MNP reference tracer with a volume of V_{ref} = 3 x 3 x 1.5 mm^3 in a grid-like manner at various selected positions in the FOV. The SF was recorded using 100 averages and background measurement subtraction at a resolution of 45 x 27 x 15 voxels and 1.5 x 1.5 x 1.5 mm^3 voxel size. The recording time of the SF was 18.5 h. The SF covered a volume of 67.5 x 40.5 x 22.5 mm^3 with a comfortable overscan (see [12]) in the x- direction related to the size of the physical FOV, as the input and the output tubes of the arterial phantom were placed in this direction. For the MNP and the chosen voxel size, an image resolution of 3.7 mm was determined for using the two-voxel analysis in [13]. The dynamic flow measurements of the arterial phantoms were performed using the same field parameter as the SF measurement, however without averaging to maintain 21.5 ms temporal resolution. At the beginning of each measurement, the background signal is recorded and removed from the recorded data in a processing step during the reconstruction. The reconstructions were performed using the Kaczmarz-algorithm with Tikhonov regularization in the ParaVision 6.0 MPI software (Bruker). The applied relative regularization factor was $\lambda = 1\cdot 10^{-5}$. The frequency components used in the reconstruction process were selected by a minimum signal-to-noise-ratio (SNR) threshold based on the SNR calculated for all frequency components in the SF. An SNR threshold of 6 was applied and components below 40 kHz were discarded. All

reconstructions were calculated with 20 Kaczmarz iterations to enforce high resolution imaging by a high number of iterations. To reduce the number of frames in the reconstruction from 4000 to 400, an averaging of 10 was applied for the phantom without the flow diverter stent because a longer observation time was needed to detect the late vortex flow in the aneurysm. We used an average factor of 2 in the experiment with flow diverter stent to reduce the number of time frames from 620 to 310. The reconstructed time frames were exported to MIPAV, the Medical Image Processing, Analysis, and Visualization application (https://mipav.cit.nih.gov/) and visualized in video sequences.

III. Results and discussion

MNP used as probes were capable to resolve the flow properties in the region of the aneurysm and the action of diverter stent in the aneurysm. Videos with millisecond resolution could be reconstructed from the MPI data. The following images show some of the key scenes of the video sequences.

III.I. Phantom without flow diverter stent

Two typical reconstructed time frames of the arterial phantom without treatment by flow diverter stent are depicted in Fig. 3. For better comparison a photograph of the corresponding phantom section is shown in the top panel of the figure. The middle panel shows the passage of the MNP at t= 9.6 s through the phantom while the late vortex flow at t = 63 s (an effect similar to the "late potentials" in MRI).

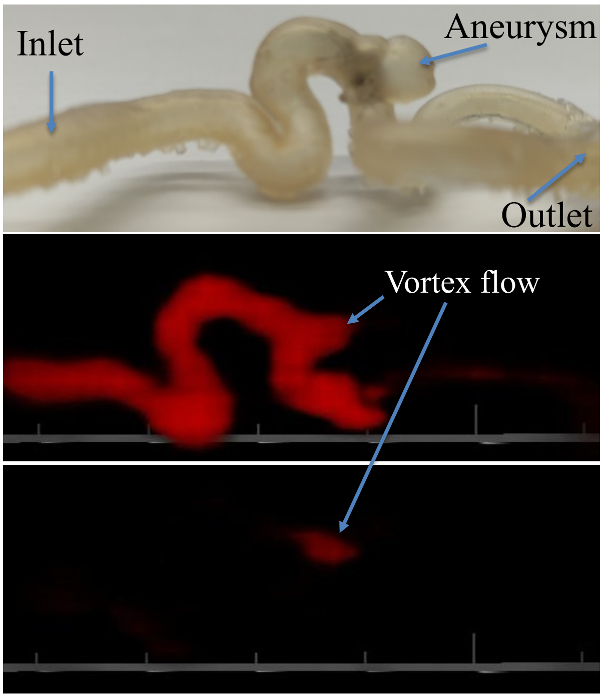

Figure 3: Imaging of the vascular phantom Top: picture of the selected section of the phantom including aneurysm, Middle: Imaged section during passage of MNP bolus, Bottom: "late potential" of the vortex flow in the aneurysm

The aneurysm represents a bulge in the vessel wall in which the trapped hemodynamic volume is set in rotation by the tangential main flow in the artery, creating the vortex flow. As the MNP bolus passes through the artery, it is also flushed into the aneurysm. While the MNP bolus in the artery is then quickly transported away, the portion in the vortex flow of the aneurysm remains much longer.

Fig. 4 gives an enlarged view of the aneurysm area showing the flow dynamics recovered by MPI. Due to the direction of flow within the artery (see Fig. 4, top), the MNP reached the lower part of the aneurysm first.

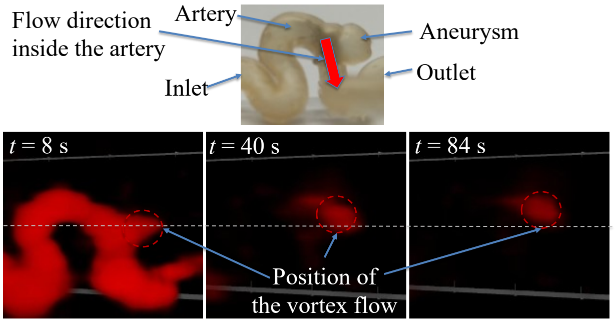

Figure 4: Flow dynamic inside the aneurysm, Top: selected section of phantom including aneurysm, Bottom: Position of the vortex flow in the aneurysm at different time steps: 8 s, 40 s and 84 s

Driven by the flow in the artery the vortex flow inside the aneurysm is changed from the bottom part (closer to the artery connection) to the upper part of the aneurysm. A dashed line has been added to the center of the bottom row of Fig. 4 to clearly mark this relative change. In addition, the images at t = 40 s and t = 84 s (outside the red dashed circle) show a small backflow from the aneurysm back into the artery. In addition, we also observe some differences in bolus outflow from different parts of the artery, which is shown in Fig. 5, and they are a function of the curvature and dilatation of the artery. In areas of strong curvature and widening of the artery, such as p1 and p2, we also observe a weak vortex formation, but much more transient than in the aneurysm. The fact that we can image this with MPI at a relatively low flow rate of 3 mL/min demonstrates the high sensitivity of this imaging modality for mapping hemodynamic flows. The simulations of the arterial flow of untreated and treated aneurysms in [14], impressively show the changes in hemodynamic flow due to treatment with a FDS, but also that the vortices and turbulence increase with increasing arterial flow rate, and this amplification should also make them easier to detect.

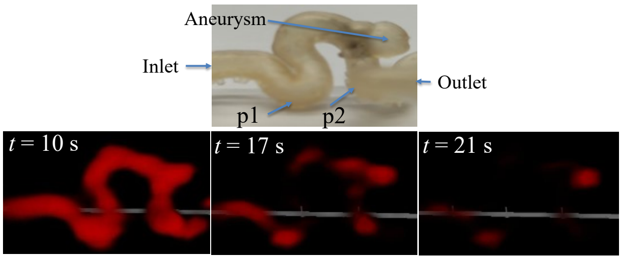

Figure 5: Outflow of the MNP bolus from different sections of the artery, Top: selected section of phantom, Bottom: Imaging of the outflow including weak vortex formations inside the curvature sections p1 and p2 at t= 10s 17s and 21 s.

III.II. Phantom with flow diverter stent

In the aneurysm phantom with flow diverter stent, the MPI reconstructions reveal that the MNP much faster pass the aneurysm region, as if there was no vortex flow to observe in the aneurysm.

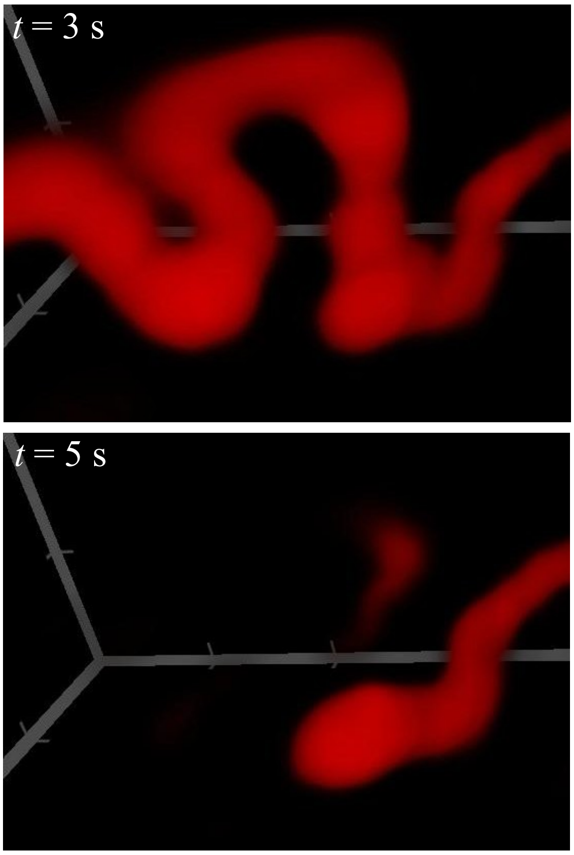

Figure 6: Reconstructed frames, Top: at 3s and Bottom: 5 s after the start of the passage of the MNP through the arterial phantom with flow diverter stent

Fig. 6 shows the MNP passage at two early time points, $t = 3$ s and $t = 5$ s, with the same selected section as in Fig. 4, but with the FDS. In contrast to the results for the aneurysm without stenting shown in III.I., we did not observe any substantial entering of arterial flow into the aneurysm, nor could vortex flow be identified. Only in a very narrow region around the mesh of the stent, the outflow of the MNP was slightly delayed as shown in Fig. 6 at $t = 5$ s. A short time interval later, all the MNP had disappeared.

Despite the diagnostic benefit of MPI flow imaging, which is certainly a long-term perspective due to the present lack of clinical, human-sized MPI scanners with sufficient sensitivity, the application of MPI to assess the success of practicing endovascular surgical skills seems directly feasible even with the present preclinical MPI scanners. So, we see the following application scenarios:

1. Success verification of surgical training.

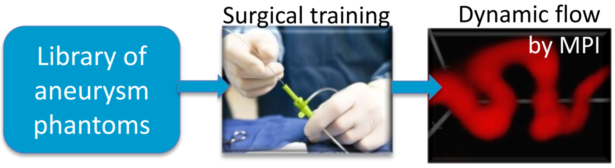

Figure 7: Configuration for training surgical practice

There are several simulation models [15] for training endovascular skills, but they all lack the ability of success verification [16], i.e., how well the desired treatment goal is achieved, and to analyze errors if necessary. Here, MPI can directly assist by imaging of changes due to the surgical intervention with sufficient temporal and spatial resolution.

2. Patient-specific selection and adaptation of FDS before surgery

Furthermore, together with 3D printing of complex patient-specific aneurysms, MPI flow imaging could be another area of application for selecting a suitable personalized flow diverter stent [17],[18] and its individual adaptation.

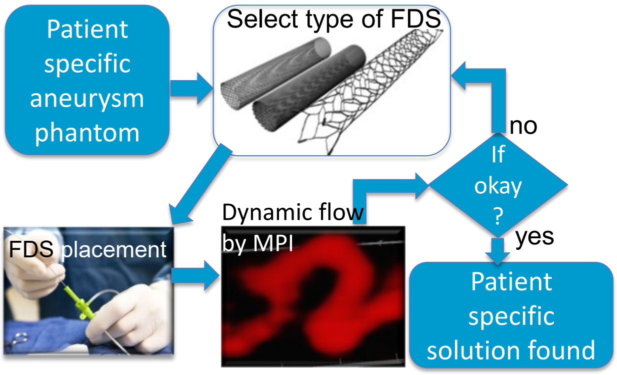

Figure 8: Configuration for personalized FDS solution

Here, the first step would be to create an aneurysm phantom from the patient's MRI aneurysm data set and then select the FDS. The next step is to adapt the stent to the aneurysm. The MPI flow analysis then determines whether the flow is directed in such a way that the flow in the aneurysm becomes stagnant. If this is not the case, a new run with a different FDS type and/or a different adaptation must be performed until the patient-specific solution is achieved. This second scenario includes the optimization of two elements by the MPI feedback, the way of placement and the type-selection of the FDS.

3. Development and optimization of new FDS designs.

This procedure would be analogous to the previous application example, yet it necessitates more detailed feedback regarding which component of the stent should be redesigned and the manner in which it should be modified, based on regional flow conditions as determined by MPI.

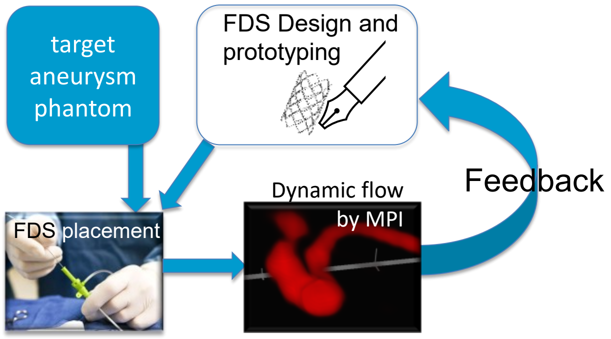

Figure 8: Configuration for new FDS design

In addition to the analysis of different flow conditions, the analysis of flow conditions for rupturing aneurysms could be expanded through the use of appropriate phantoms. Furthermore, the stents could be investigated for these conditions.

IV. Conclusions

Based on the long residence time of the MNP in the aneurysm without a flow diverter stent resolved by MPI, we conclude that a stable vortex flow was formed in the aneurysm with little fluid exchange with the artery. Furthermore, we observed some vortex flows at strong curvatures in the artery (s. Fig. 5), but these became only visible for a short time due to the higher fluid exchange rate at these locations. However, the sensitivity of MPI for the detection of such small flow turbulence is already evident. We therefore suppose that it will be possible to visualize by MPI the complex fluid mechanics of a wide-neck aneurysm and other variations, as well. The detection of the small flow at the mesh of the flow diverter stent (s. Fig. 6, $t = 5$ s) already demonstrates the high performance of the MPI and opens the possibility of achieving even more complete suppression of the flow in the aneurysm by using a different stent variant with, for example, narrower meshes. In total, already here the powerful application of MPI to visualize many details of the fluid mechanics in an aneurysm has been successfully demonstrated. In addition to the potential use of this imaging as feedback for training in endovascular surgical skills and the individualization of flow diverter solutions for personalized treatment of complex aneurysms, there is another application scenario where this feedback can be used to optimize stent designs for different endovascular treatments.

We will continue our work with other more complex aneurysms like fussy-form or wide-neck aneurysms and with a more detailed setup for several realistic flow conditions, like systolic and diastolic flow.

Author's Statement

The authors declare that they have no known competing financial interests or personal relationships that could have appeared to influence the work reported in this paper.

Informed consent: Informed consent has been obtained from all individuals included in this study.

Ethical approval: The research related to human use complies with all the relevant national regulations, institutional policies and was performed in accordance with the tenets of the Helsinki Declaration, and has been approved by the authors' institutional review board or equivalent committee.

References

[1] T. Willis, *Anatomy of the Brain and Nerves*. MQUP, 1965. doi: 10.2307/j.ctt1t88wg2.

[2] K. D. Dennis, T. L. Rossman, D. F. Kallmes, and D. Dragomir-Daescu, "Intra-aneurysmal flow rates are reduced by two flow diverters: an experiment using tomographic particle image velocimetry in an aneurysm model", doi: 10.1136/neurintsurg-2014-011323.

[3] Y. J. Alderazi, D. Shastri, T. Kass-Hout, C. J. Prestigiacomo, and C. D. Gandhi, "Flow diverters for intracranial aneurysms," *Stroke Res. Treat.*, vol. 2014, 2014, doi: 10.1155/2014/415653.

[4] T. Becske *et al.*, "Pipeline for Uncoilable or Failed Aneurysms: Results from a Multicenter Clinical Trial," *Radiology*, vol. 267, no. 3, pp. 858–868, Jun. 2013, doi: 10.1148/radiol.13120099.

[5] J. Sedlacik *et al.*, "Magnetic particle imaging for high temporal resolution assessment of aneurysm hemodynamics," *PLoS One*, 2016, doi: 10.1371/journal.pone.0160097.

[6] M. Graeser *et al.*, "Human-sized magnetic particle imaging for brain applications," *Nat. Commun.*, vol. 10, no. 1, 2019, doi: 10.1038/s41467-019-09704-x.

[7] A. Remmo, O. Kosch, L. Kampen, A. Ludwig, F. Wiekhorst, and N. Löwa, "Counting cells in motion by quantitative real-time magnetic particle imaging," *Sci. Reports 2024 141*, vol. 14, no. 1, pp. 1–10, 2024, doi: 10.1038/s41598-024-54784-5.

[8] M. Herzberg *et al.*, "Magnetic particle imaging for artifact-free imaging of intracranial flow diverter stents: A phantom study," *Phys. Medica*, vol. 88, no. June, pp. 65–70, 2021, doi: 10.1016/j.ejmp.2021.06.018.

[9] D. S. Shin, C. P. Carroll, M. Elghareeb, B. L. Hoh, and B. T. Kim, "The evolution of flow-diverting stents for cerebral aneurysms; historical review, modern application, complications, and future direction," *J. Korean Neurosurg. Soc.*, vol. 63, no. 2, pp. 137–152, 2020, doi: 10.3340/jkns.2020.0034.

[10] H. Paysen *et al.*, "Improved sensitivity and limit-of-detection using a receive-only coil in magnetic particle imaging," *Phys. Med. Biol.*, vol. 63, no. 13, p. 13NT02, Jul. 2018, doi: 10.1088/1361-6560/aacb87.

[11] J. Rahmer, J. Weizenecker, B. Gleich, and J. Borgert, "Analysis of a {3-D} system function measured for magnetic particle imaging," *IEEE Trans. Med. Imaging*, vol. 31, no. 6, pp. 1289–1299, 2012, doi: 10.1109/TMI.2012.2188639.

[12] A. Weber, F. Werner, J. Weizenecker, T. M. Buzug, and T. Knopp, "Artifact free reconstruction with the system matrix approach by overscanning the field-free-point trajectory in magnetic particle imaging," *Phys. Med. Biol.*, vol. 61, no. 2, pp. 475–487, 2016, doi: 10.1088/0031-9155/61/2/475.

[13] O. Kosch, A. Remmo, J. Wells, and F. Wiekhorst, "Two-voxel-analysis to assess the spatial resolution of a tracer in Lissajous-scanning magnetic particle imaging," *J. Magn. Magn. Mater.*, vol. 565, no. July 2022, p. 170170, 2023, doi: 10.1016/j.jmmm.2022.170170.

[14] H. G. Morales *et al.*, "Does arterial flow rate affect the assessment of flow-diverter stent performance?," *Am. J. Neuroradiol.*, vol. 37, no. 12, pp. 2293–2298, 2016, doi: 10.3174/ajnr.A4933.

[15] S. K. Neequaye, R. Aggarwal, I. Van Herzeele, A. Darzi, and N. J. Cheshire, "Endovascular skills training and assessment," *J. Vasc. Surg.*, vol. 46, no. 5, pp. 1055–1064, 2007, doi: 10.1016/j.jvs.2007.05.041.

[16] S. Kumarapuram, S., Sreenivasan, S. Roychowdhury and G. Nanda, A., Khandelwal, P., Sonig, A., Gupta, "The advent of endovascular neurosurgery, its impact on neurosurgery residency training, and the making of a vascular neurosurgeon," *JNS*, vol. 139, no. 1, pp. 292–297, 2023, [Online]. Available: https://doi.org/10.3171/2023.1.JNS222805

[17] D. Z.-F. and Y. V. T. W. Peach, M. Ngoepe, K. Spranger, "Personalizing flow-diverter intervention for cerebral aneurysms: from computational hemodynamics to biochemical modeling," *Int. J. Numer. Meth. Biomed. Engng.*, vol. 30, no. 11, pp. 1387–1407, 2014, doi: 10.1002/cnm.2663.

[18] J. Kuhl, A. Ding, N. T. Ngo, A. Braschkat, J. Fiehler, and D. Krause, "Design of Personalized Devices—The Tradeoff between Individual Value and Personalization Workload," *Appl. Sci.*, vol. 11, no. 1, pp. 1–21, 2021, doi:

10.3390/app11010241.
